# KBN2201 Attenuates High-Fat Diet-Induced Adipose Tissue Expansion and Body Weight Gain in Male Mice

**DOI:** 10.3390/ijms27146155

**Published:** 2026-07-09

**Authors:** Moonhang Kim, Jeong-Hyeon Heo, Seok-Hwan Chang, Sun-Young Lee, Jihun Kim, Chaeeun Park, Youjin Lee, Moon-Geun Shin, Jong Sung Kim, Mi Ran Choi, Sang-Rae Lee

**Affiliations:** 1 Efficacy Test Center for Mental & Behavioral Disorders, Ajou University Hospital, Suwon 16499, Republic of Korea; mook1052@ajou.ac.kr (M.K.); tjrghksekrzj@ajou.ac.kr (S.-H.C.); sun02lee@ajou.ac.kr (S.-Y.L.); mgshin@ajou.ac.kr (M.-G.S.); kjs0829@ajou.ac.kr (J.S.K.); 2 Department of Pharmacology, Ajou University School of Medicine, Suwon 16499, Republic of Korea; hjh2g@ajou.ac.kr (J.-H.H.); soulmate415@ajou.ac.kr (J.K.); smilechanni@ajou.ac.kr (C.P.); youjin1219@ajou.ac.kr (Y.L.); 3 BK21 R&E Initiative for Advanced Precision Medicine, Ajou University, Suwon 16499, Republic of Korea; 4 Department of Biomedical Sciences, Graduate School, Ajou University, Suwon 16499, Republic of Korea; 5 Laboratory Animal Research Center, Ajou University School of Medicine, Suwon 16499, Republic of Korea

**Keywords:** KBN2201, high-fat diet, obesity, adipose tissue, food intake, *Pyy*

## Abstract

Obesity is characterized by pathological adipose tissue expansion and dysregulated energy homeostasis. In this study, we examined whether KBN2201 attenuates high-fat diet (HFD)-induced obesity-associated phenotypes and whether these effects are accompanied by changes in appetite-regulatory markers. C57BL/6J mice were fed a normal chow diet or HFD and orally administered vehicle or KBN2201 (20 mg/kg/day) for 12 weeks. In male mice, KBN2201 reduced HFD-induced body weight gain, adipose tissue accumulation, and adipocyte hypertrophy. KBN2201 was also associated with lower cage-level caloric intake under HFD-fed conditions. In epididymal white adipose tissue (eWAT), KBN2201 suppressed *Srebf1* expression and reduced HFD-induced increases in *Tnf* and *Tgfb1* expression. In the colon, KBN2201 further increased *Pyy* mRNA expression, and a positive mRNA-level association was observed between colonic *Pyy* and *Npy2r* expression in NTS-containing brainstem tissue. Behavioral analyses provided no evidence of overt locomotor suppression under the present experimental conditions. These findings suggest that KBN2201 attenuates HFD-induced obesity-associated phenotypes in male mice, accompanied by a lower cage-level food intake pattern, reduced adipose tissue expansion, and changes in the expression of colonic *Pyy* and NTS-containing brainstem *Npy2r*.

## 1. Introduction

Obesity is a complex and chronic metabolic disorder characterized by excessive adiposity, pathological adipose tissue expansion, and disrupted energy homeostasis. Its hallmark features include abnormal lipid accumulation, adipose tissue remodeling, and chronic low-grade inflammation [1,2]. This condition generally develops from a sustained imbalance between energy intake and expenditure and is shaped by a complex interplay of nutritional, environmental, endocrine, and genetic factors [3].

Food intake is regulated by coordinated interactions between peripheral metabolic signals and central neural circuits [4,5]. Although the hypothalamus serves as a primary hub for homeostatic appetite control through orexigenic neuropeptide Y (NPY)/agouti-related peptide (AgRP) and anorexigenic pro-opiomelanocortin (POMC)/cocaine- and amphetamine-regulated transcript (CART) neurons [6], feeding regulation extends beyond this classical axis and involves distinct neuronal subpopulations as well as extra-hypothalamic circuits, including brainstem and limbic pathways, that integrate energy status, palatability, and emotional context [7,8,9]. Within this network, the gut–brain axis serves as a major pathway for transmitting nutrient-derived satiety signals from the intestine to the central nervous system [10,11]. Postprandial peptide YY (PYY), particularly its active form PYY3–36, preferentially activates Y2 receptor (Y2R)-mediated signaling to suppress food intake, and this anorexigenic effect may involve both endocrine actions and vagal afferent pathways that relay satiety information to brainstem nuclei such as the nucleus tractus solitarius (NTS) [10,12]. Accordingly, assessing colonic PYY expression together with central feeding-related markers such as Y2R, AgRP, and NPY may provide insight into gut–brain appetite-regulatory changes associated with altered food intake [6,11,12,13].

The clinical success of gut hormone-based therapies, including glucagon-like peptide-1 (GLP-1) receptor agonists and dual glucose-dependent insulinotropic peptide (GIP)/GLP-1 receptor agonists, further validates the therapeutic potential of targeting these pathways [14,15]. However, remaining challenges, including the need for long-term treatment, injectable formulations for many high-efficacy therapies, gastrointestinal adverse effects, and weight regain after discontinuation, underscore the need for novel orally administrable anti-obesity strategies that modulate energy intake without compromising physical activity or exacerbating behavioral stress [16,17].

High-fat diet (HFD)-induced rodent models are widely used to investigate obesity-associated metabolic pathology because they recapitulate key features of human obesity, including body weight gain, adipocyte hypertrophy, increased adiposity, insulin resistance, adipose tissue remodeling, and inflammatory signaling [18,19,20]. Sex is also an important biological variable in metabolic regulation, and male and female rodents can differ in their susceptibility to HFD-induced obesity, fat mass accumulation, and metabolic dysfunction [21,22]. These differences have been associated with sex hormone-dependent regulation of adipocyte function, insulin sensitivity, and energy homeostasis, supporting the need to evaluate treatment responses in both sexes [22,23]. Together, these inflammatory, remodeling, and sex-dependent metabolic features indicate that HFD-induced obesity represents a chronic pathological condition involving progressive disruption of systemic homeostasis.

KBN2201, a small-molecule derivative of 2-((2-oxopropanoyl)oxy)-4-(trifluoromethyl)benzoic acid, has previously demonstrated neuroprotective and anti-inflammatory activity in the postischemic rat brain and in a late-stage degenerative disease mouse model characterized by chronic inflammatory pathology [24,25]. In these contexts, KBN2201 attenuated disease-associated pathological protein accumulation, neuronal injury, and inflammatory responses while promoting protective and regeneration-related changes. Although these findings suggest that KBN2201 may modulate inflammatory and tissue remodeling processes, its potential role in metabolic disease remains undefined.

We hypothesized that KBN2201 would attenuate HFD-induced obesity-associated phenotypes, particularly adipose tissue expansion and adipocyte hypertrophy, and that these effects would be associated with changes in feeding-related metabolic responses and gut–brain appetite-regulatory markers. To address this hypothesis, we evaluated body weight gain, adipose tissue accumulation, adipocyte hypertrophy, lipogenic remodeling, inflammatory and remodeling-related gene expression, and cage-level food intake patterns in HFD-fed mice treated with KBN2201. We also explored whether cage-level food intake patterns were associated with gut–brain appetite-regulatory markers, including colonic *Pyy* expression and feeding-related gene expression in the NTS and hypothalamus.

## 2. Results

### 2.1. KBN2201 Attenuates HFD-Induced Body Weight Gain and Adipose Tissue Accumulation

**Figure 1:**
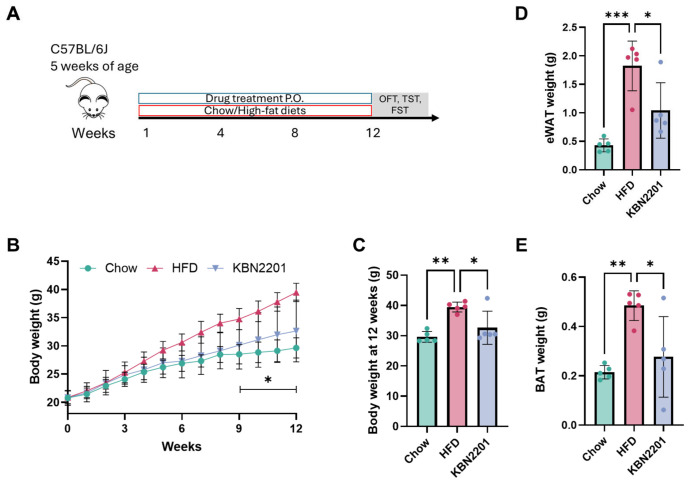
Experimental design, body weight changes, and adipose tissue weights in male high-fat diet (HFD)-fed mice treated with KBN2201. (**A**) Experimental scheme. Five-week-old male and female C57BL/6J mice were fed either a chow diet or a HFD and orally administered vehicle or KBN2201 for 12 weeks. Behavioral tests, including the open field test (OFT), tail suspension test (TST), and forced swim test (FST), were performed at the end of the experimental period. (**B**) Weekly body weight changes in male mice during the 12-week experimental period. (**C**) Body weight of male mice at week 12. (**D**) Epididymal white adipose tissue (eWAT) weight in male mice at the end of the experiment. (**E**) Brown adipose tissue (BAT) weight in male mice at the end of the experiment. Data are presented as the mean ± SEM. Body weight changes over time were analyzed by two-way repeated-measures ANOVA followed by Tukey’s multiple comparisons test. Endpoint body weight and tissue weights were analyzed by one-way ANOVA followed by Tukey’s multiple comparisons test. Statistical significance is indicated by horizontal bars or symbols: * *p* < 0.05, ** *p* < 0.01 and *** *p* < 0.001. *n* = 5 mice per group.

To evaluate the effect of KBN2201 on HFD-induced obesity-associated phenotypes, body weight changes and adipose tissue weights were assessed in male mice assigned to the normal chow diet plus vehicle control group (Chow), HFD plus vehicle control group (HFD), or HFD plus KBN2201 group (KBN2201). HFD-fed male mice showed a progressive increase in body weight compared with chow-fed controls. KBN2201 treatment significantly reduced body weight gain relative to the HFD group from week 9 onward (*p* < 0.05; Figure 1B). At week 12, body weight in the HFD group was approximately 36% higher than that in the chow group (*p* < 0.01), whereas KBN2201 treatment reduced body weight by approximately 18% compared with the HFD group (*p* < 0.05; Figure 1C). In female mice, HFD feeding also increased body weight over the 12-week experimental period compared with chow-fed controls; however, KBN2201 did not significantly reduce final body weight compared with the HFD group (Figure S1A).

Consistent with the male body weight results, adipose tissue weights were also markedly altered in male mice. HFD increased epididymal white adipose tissue (eWAT) weight to approximately 1.8 g, corresponding to an increase of about 311% relative to the chow group, while KBN2201 treatment reduced eWAT weight by approximately 43% compared with the HFD group (Figure 1D). Similarly, brown adipose tissue (BAT) weight was increased by approximately 129% in the HFD group relative to the chow group, and KBN2201 treatment decreased BAT weight by approximately 44% compared with the HFD group (Figure 1E). Additional tissue weight-to-body weight ratio analysis in male mice showed that HFD feeding reduced the relative weights of tibialis anterior (TA) muscle and colon compared with the chow group, whereas KBN2201 treatment increased the rectus femoris weight-to-body weight ratio compared with the HFD group (Figure S1B).

### 2.2. KBN2201 Is Associated with Lower Cage-Level Caloric and Food Intake in Male HFD-Fed Mice

**Figure 2:**
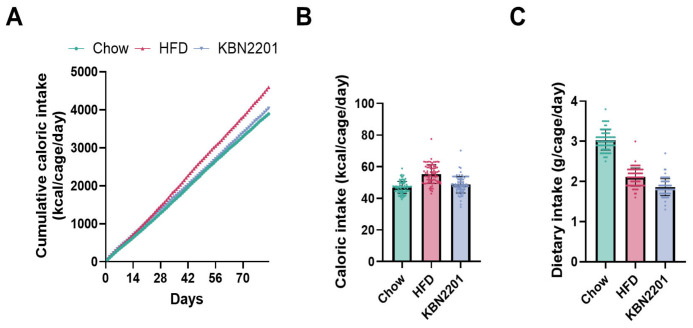
Cage-level caloric intake and food intake in male high-fat diet (HFD)-fed mice treated with KBN2201. (**A**) Cumulative cage-level energy intake in male mice during the experimental period. (**B**) Daily cage-level caloric intake in male mice. (**C**) Daily cage-level food intake in male mice, calculated based on food weight. Food intake was measured at the cage level, with one cage per group and five mice per cage; therefore, these data are presented descriptively without inferential statistical testing. Data are presented as the mean ± SEM of repeated cage-level measurements.

To determine whether the KBN2201-associated reduction in body weight gain was accompanied by altered feeding patterns, cage-level caloric intake and food intake were analyzed. The cumulative caloric intake curve showed that the HFD group exhibited the highest caloric intake over the experimental period, whereas the male KBN2201-treated group displayed a lower cumulative caloric intake pattern that was closer to the chow group than to the HFD group (Figure 2A). Consistent with this pattern, daily cage-level caloric intake was higher in the male HFD group than in the chow group, whereas the male KBN2201-treated group showed lower daily cage-level caloric intake than the HFD group (Figure 2B). In addition, daily food intake by weight showed a lower pattern in the male KBN2201-treated group than in the HFD group (Figure 2C), indicating that KBN2201 treatment was associated with a lower cage-level food intake pattern under HFD-fed conditions in male mice. In female mice, HFD-fed groups showed higher cage-level caloric intake than chow-fed controls, whereas KBN2201 did not clearly reduce cumulative caloric intake, daily caloric intake, or daily food intake compared with the female HFD group (Figure S1C).

### 2.3. KBN2201 Treatment Is Accompanied by Changes in Gut–Brain Appetite-Regulatory Gene Expression in Male HFD-Fed Mice

**Figure 3:**
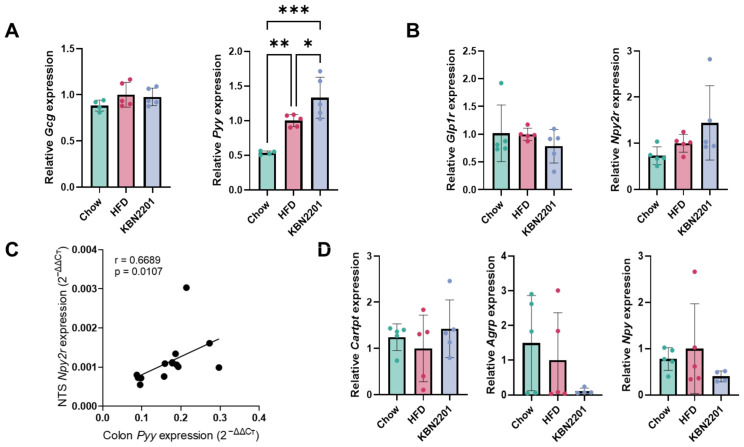
Gut and brain appetite-regulatory gene expression in male high-fat diet (HFD)-fed mice treated with KBN2201. (**A**) Relative mRNA expression of *Gcg* and *Pyy* in the colon of male mice. (**B**) Relative mRNA expression of *Glp1r* and *Npy2r* in brainstem samples containing the nucleus tractus solitarius (NTS). (**C**) Correlation analysis between colonic *Pyy* expression and brainstem *Npy2r* expression. (**D**) Relative mRNA expression of *Cartpt*, *Agrp*, and *Npy* in the hypothalamus. Data are presented as the mean ± SEM. Gene expression among groups was analyzed by one-way ANOVA followed by Tukey’s multiple comparisons test. Correlation analysis was performed using Pearson’s correlation test. Statistical significance is indicated by horizontal bars or symbols: * *p* < 0.05, ** *p* < 0.01 and *** *p* < 0.001. Individual dots represent individual mice. *n* = 5 mice per group.

To examine whether the lower cage-level food intake pattern observed in male KBN2201-treated mice was accompanied by changes in gut–brain appetite-regulatory markers, the expression of appetite-related hormones and receptors was analyzed in the colon, NTS-containing brainstem tissue, and hypothalamus. In the colon, *Gcg*, which encodes proglucagon-derived peptides including GLP-1, was not altered among groups, whereas *Pyy* mRNA expression was significantly increased in the male HFD group compared with the chow group (approximately 1.9-fold, *p* < 0.01) and was further elevated in the male KBN2201-treated group relative to the male HFD group (approximately 1.3-fold, *p* < 0.05; Figure 3A). We next examined the expression of corresponding receptor genes in NTS-containing brainstem tissue. While *Glp1r* mRNA expression did not differ among groups, *Npy2r* mRNA expression was numerically higher in the male KBN2201-treated group, but this difference did not reach statistical significance (Figure 3B). Correlation analysis showed a positive mRNA-level association between colonic *Pyy* and *Npy2r* in NTS-containing brainstem tissue (r = 0.6689, *p* = 0.0107; Figure 3C). In the hypothalamus, expression of the appetite-regulatory neuropeptides *Cartpt*, *Agrp*, and *Npy* did not show statistically significant differences among groups (Figure 3D). Nevertheless, the orexigenic neuropeptides *Agrp* and *Npy* were numerically lower in the male KBN2201-treated group than in the male HFD group, without reaching statistical significance.

### 2.4. KBN2201 Attenuates Adipocyte Hypertrophy and Suppresses Lipogenic Remodeling-Related Gene Expression in eWAT

**Figure 4:**
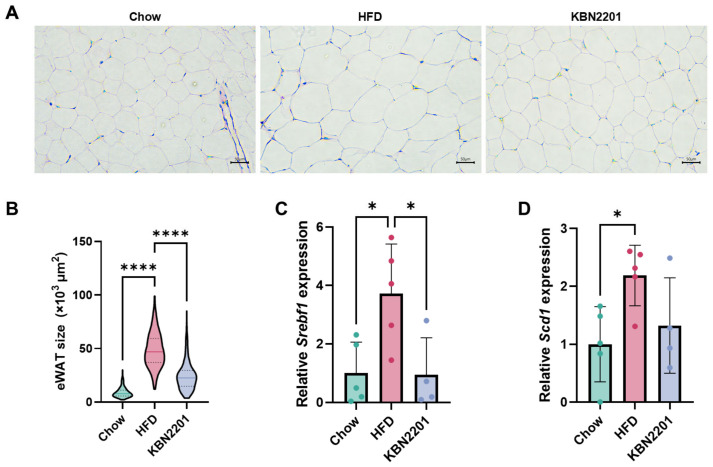
Adipocyte morphology and lipogenesis-related gene expression in male epididymal white adipose tissue (eWAT) from high-fat diet (HFD)-fed mice treated with KBN2201. (**A**) Representative hematoxylin and eosin (H&E)-stained images of eWAT from male mice in the chow, HFD, and KBN2201 groups. Scale bar, 50 μm. (**B**) Adipocyte size distribution is shown for visualization, and statistical analysis was performed using the mean adipocyte area of each mouse as the biological unit. The violet line indicates the mean, and the dotted lines indicate the SEM. (**C**) Relative mRNA expression of *Srebf1* in male eWAT. (**D**) Relative mRNA expression of *Scd1* in male eWAT. Data are presented as the mean ± SEM. Gene expression data were analyzed by one-way ANOVA followed by Tukey’s multiple comparisons test. Statistical significance is indicated by horizontal bars or symbols: * *p* < 0.05 and **** *p* < 0.0001. Individual dots represent individual mice. *n* = 4–5 mice per group.

To determine whether KBN2201 affects adipose tissue morphology in male HFD-fed mice, eWAT tissue sections were examined by hematoxylin & eosin (H&E) staining. Adipocytes from male HFD-fed mice were visibly enlarged compared with those from male chow-fed mice, whereas KBN2201 treatment reduced adipocyte size relative to the male HFD group (Figure 4A). Quantitative analysis confirmed that HFD feeding markedly increased adipocyte size, while KBN2201 treatment significantly decreased adipocyte size compared with the HFD group (Figure 4B).

To further assess whether these morphological changes were associated with altered lipogenic gene expression in male eWAT, representative lipogenesis-related markers were analyzed. *Srebf1*, a key transcriptional regulator of lipogenesis and adipogenesis, was significantly increased in the male HFD group compared with the male chow group, and this increase was significantly suppressed by KBN2201 treatment (Figure 4C). In addition, *Scd1* expression was elevated in the male HFD group relative to the male chow group. Although *Scd1* expression was numerically lower in the KBN2201-treated group than in the HFD group, the difference was not statistically significant (Figure 4D).

Because KBN2201 reduced BAT weight in male HFD-fed mice, thermogenic gene expression was also examined in male BAT. Relative *Ucp1* and *Cidea* mRNA expression levels were not significantly altered among the chow, HFD, and KBN2201-treated groups (Figure S1D), suggesting that the reduction in BAT mass was not accompanied by clear transcriptional activation of canonical BAT thermogenic markers.

### 2.5. KBN2201 Attenuates HFD-Induced Increases in Tnf and Tgfb1 Expression in Male Adipose Tissue

**Figure 5:**
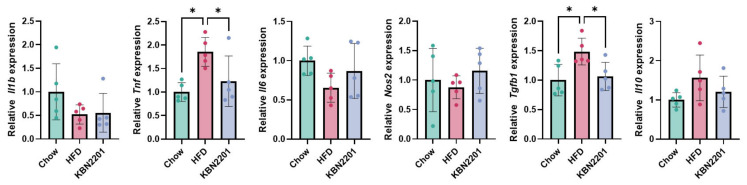
Inflammation- and remodeling-related gene expression in male epididymal white adipose tissue (eWAT). Relative mRNA expression levels of inflammation- and remodeling-related markers were analyzed in eWAT from male mice in the chow, high-fat diet (HFD), and KBN2201 groups. The analyzed genes included *Il1b*, *Tnf*, *Il6*, *Nos2*, *Tgfb1*, and *Il10*. KBN2201 selectively reduced HFD-induced increases in *Tnf* and *Tgfb1* expression in male eWAT, whereas *Il1b*, *Il6*, *Nos2*, and *Il10* expression were not significantly altered among groups. Data are presented as the mean ± SEM. Statistical analysis was performed using one-way ANOVA followed by Tukey’s multiple comparisons test. Statistical significance is indicated by horizontal bars: * *p* < 0.05. Individual dots represent individual mice. *n* = 5 mice per group.

To assess whether KBN2201 affects inflammatory and remodeling-related responses in adipose tissue, the mRNA expression levels of selected genes were analyzed in male eWAT (Figure 5). *Il1b*, *Il6*, and *Nos2* expression did not show significant differences among the chow, HFD, and KBN2201-treated groups. In contrast, *Tnf* expression was significantly increased in the HFD group compared with the chow group, and this increase was significantly reduced by KBN2201 treatment. Similarly, *Tgfb1* expression was significantly increased in the HFD group compared with the chow group, whereas KBN2201 treatment significantly reduced *Tgfb1* expression compared with the HFD group. *Il10* expression was not significantly changed among groups.

### 2.6. Behavioral Assessment of Locomotor Activity and Stress-Related Responses in HFD-Fed Mice

**Figure 6:**
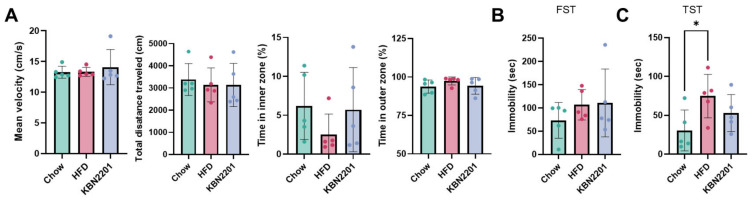
Behavioral analyses in male high-fat diet (HFD)-fed mice treated with KBN2201. (**A**) Open field test (OFT) parameters in male mice, including mean velocity, total distance traveled, and percentage of time spent in the inner/outer zone. (**B**) Immobility time in the forced swim test (FST) in male mice. (**C**) Immobility time in the tail suspension test (TST) in male mice. Data are presented as the mean ± SEM. Statistical analysis was performed using one-way ANOVA followed by Tukey’s multiple comparisons test. Statistical significance is indicated by horizontal bars: * *p* < 0.05. Individual dots represent individual mice. *n* = 5 mice per group.

To determine whether the lower cage-level food intake pattern and reduced body weight observed in male KBN2201-treated mice could be explained by nonspecific behavioral suppression, locomotor activity, anxiety-like behavior, and depression-like behavior were assessed in male mice. In the open field test (OFT), mean velocity and total distance traveled were not significantly different among the chow, HFD, and KBN2201-treated groups. In addition, the percentage of time spent in the inner/outer zone did not differ significantly among groups, suggesting no clear alteration in open-field zone preference under the present experimental conditions (Figure 6A).

In the forced swim test (FST), immobility time was not significantly altered by HFD feeding or KBN2201 treatment (Figure 6B). In contrast, the tail suspension test (TST) showed that HFD feeding significantly increased immobility time compared with the chow group, indicating an HFD-associated increase in depression-like or stress-related behavioral response (Figure 6C). KBN2201-treated mice showed a non-significant reduction in immobility time compared with the HFD group (Figure 6C).

## 3. Discussion

Obesity is a major metabolic health challenge characterized by pathological adipose tissue expansion, dysregulated energy homeostasis, and chronic low-grade inflammation [1,2,3]. HFD-induced rodent models are widely used to investigate obesity-associated metabolic pathology because they recapitulate key features of human obesity, including body weight gain, adipocyte hypertrophy, increased adiposity, inflammatory signaling, and adipose tissue remodeling [18,19,20]. Although the clinical success of gut hormone-based therapies supports the therapeutic potential of appetite-regulatory pathways [14,15], there remains a need for orally administered agents that attenuate adipose tissue expansion and modulate energy intake without causing nonspecific behavioral suppression or stress-related adverse effects [16,17]. In this context, the present study evaluated whether KBN2201 modulates HFD-induced obesity-associated phenotypes and related metabolic regulatory markers. Overall, our findings indicate that KBN2201-associated effects were not uniform across sexes but were most evident in male HFD-fed mice. Therefore, the following discussion focuses on the sex-biased response pattern, the possible contribution of food intake and gut–brain satiety-related gene expression, and adipose tissue remodeling under HFD-fed conditions.

The present study showed a sex-biased response pattern to KBN2201, with the main KBN2201-associated effects observed in male HFD-fed mice. This pattern is consistent with a previous study showing that male and female C57BL/6J mice differ in their metabolic responses to HFD or Western diet exposure, including body weight gain, food intake, locomotor activity, energy expenditure, glucose tolerance, and insulin sensitivity [26]. Ovarian estrogen signaling also regulates energy homeostasis by suppressing food intake, increasing energy expenditure, and modulating fat distribution through estrogen receptor-α-dependent actions in hypothalamic neuronal populations, including POMC and SF1 neurons [27,28,29]. Thus, endogenous estrogen-dependent metabolic regulation may have reduced the detectable additional effect of KBN2201 in female mice. Sex-dependent pharmacokinetic or pharmacodynamic differences may also have contributed to the weaker response in females [30,31]. However, estrous cycle stage, circulating sex hormone levels, KBN2201 exposure, and tissue distribution were not assessed. Therefore, the sex-biased response pattern observed here requires further validation using hormonal and sex-specific pharmacokinetic analyses.

In this study, KBN2201-treated male HFD-fed mice showed a lower cage-level food intake pattern together with increased colonic *Pyy* expression and a numerical increase in *Npy2r* expression in NTS-containing brainstem tissue. Previous studies have shown that PYY3–36 functions as a gut-derived anorexigenic peptide and can suppress food intake through Y2R-dependent mechanisms involving hypothalamic and hindbrain appetite-regulatory regions, including the NTS [13,32]. In this context, the increase in colonic *Pyy* expression and its positive correlation with *Npy2r* expression in NTS-containing brainstem tissue may indicate an association between intestinal satiety-related signaling and brainstem appetite-related gene-expression changes. However, because the present data are limited to gene-expression changes and correlation analysis, these findings should be interpreted as exploratory associations rather than evidence of functional activation of the PYY–Y2R pathway.

KBN2201 also attenuated HFD-induced adipose tissue expansion in male mice, as shown by reduced adipocyte hypertrophy and altered remodeling-related gene expression. This finding is consistent with previous studies showing that adipocyte hypertrophy is closely associated with adipose tissue dysfunction, local hypoxia, inflammatory activation, extracellular matrix remodeling, and metabolic stress in obesity [33,34,35,36,37]. In addition, reduced *Srebf1* expression may reflect attenuation of HFD-associated lipogenic remodeling, given the role of SREBP-1 in adipocyte lipogenesis and lipid metabolic regulation [38,39]. KBN2201 also reduced *Tnf* and *Tgfb1* expression, suggesting attenuation of selected inflammatory and remodeling-associated responses. TNF-α is a representative mediator of obesity-associated adipose inflammatory dysfunction [40], whereas TGF-β signaling is closely linked to extracellular matrix deposition, adipose tissue fibrosis, impaired adipose tissue expandability, and metabolic dysfunction [41,42]. Thus, the downregulation of *Tgfb1* is more appropriately interpreted as a change related to pathological fibrotic remodeling rather than as evidence of broad immune regulation. Because *Il1b*, *Il6*, *Nos2*, and *Il10* were not significantly altered, the present data do not support generalized anti-inflammatory activity. Thus, these adipose tissue findings are best interpreted as KBN2201-associated attenuation of adipocyte hypertrophy, lipogenic remodeling, and selected fibrotic/remodeling-associated markers under HFD-fed conditions. However, because KBN2201-treated mice also showed lower body weight and reduced adiposity, these changes may partly reflect secondary consequences of reduced adiposity rather than direct adipose tissue-specific actions of KBN2201.

Our behavioral analyses indicate that the lower cage-level food intake pattern observed in KBN2201-treated male HFD-fed mice was not accompanied by overt nonspecific behavioral suppression. This assessment is relevant because HFD exposure and obesity-associated metabolic stress have been reported to influence emotional, stress-related, and depression-like behavioral responses in rodents [43,44,45]. In the present study, KBN2201-treated male mice did not show reduced locomotor activity in the OFT, implying that the lower cage-level food intake pattern was not attributable to impaired mobility. In addition, KBN2201 did not exacerbate anxiety-like or stress-related behavioral responses under HFD-fed conditions. Although HFD feeding increased immobility time in the TST, KBN2201-treated male mice showed only a non-significant reduction in immobility compared with the HFD group. Taken together, these findings provide limited behavioral context indicating that the lower cage-level food intake pattern observed in KBN2201-treated HFD-fed mice was not accompanied by overt changes in gross locomotor activity or severe stress-related behavioral indices. However, these assays do not assess food palatability, motivation to eat, malaise-like responses, or diet-independent drug effects under chow-fed conditions; therefore, direct appetite-regulatory mechanisms remain to be established.

This study has several limitations. First, although both sexes were included, the main KBN2201-associated effects were observed primarily in male mice; therefore, the findings should not be generalized across sexes without further validation. Interpretation of this sex-biased response pattern is limited by the lack of estrous cycle staging, circulating estrogen measurement, and sex-specific pharmacokinetic profiling. Second, the present design did not include a chow + KBN2201 group, pair-fed controls, or weight-matched controls. Therefore, this study cannot determine whether KBN2201 has diet-independent effects under chow-fed conditions or whether the adipose tissue changes reflect direct adipose tissue-specific actions of KBN2201 rather than secondary consequences of lower body weight, reduced adiposity, or lower cage-level intake. In addition, food intake was measured at the cage level, with one cage per group for each sex; therefore, food intake and caloric intake data should be interpreted as descriptive cage-level repeated measurements rather than independent biological replicates. Third, the present study did not directly validate the functional involvement of PYY–Y2R signaling. Although colonic *Pyy* expression was increased, circulating PYY protein levels, active PYY3–36, Y2R-dependent signaling, receptor blockade, and NTS neuronal activation markers such as c-Fos were not assessed. Moreover, *Npy2r* expression was measured in NTS-containing brainstem tissue rather than purified NTS tissue or isolated NTS neuronal populations, limiting anatomical and cell-type-specific interpretation. Therefore, the observed changes in colonic *Pyy* and brainstem *Npy2r* expression should be interpreted as associative gut–brain appetite-related markers. Finally, because body weight regulation depends on both energy intake and energy expenditure [46,47], energy expenditure, substrate utilization, and thermogenic activity were not directly measured. Additional metabolic cage analyses are needed to determine whether KBN2201 affects energy expenditure, substrate utilization, or whole-body energy metabolism.

In conclusion, KBN2201 attenuated HFD-induced obesity-associated phenotypes primarily in male mice. These effects were associated with a lower cage-level food intake pattern, reduced adipose tissue expansion, and changes in colonic *Pyy* and brainstem *Npy2r* expression. Although causal involvement of the PYY–Y2R pathway was not established, these findings support further investigation of KBN2201 as a potential modulator of HFD-induced obesity-associated phenotypes.

## 4. Materials and Methods

### 4.1. Animal Model of HFD-Induced Obesity

Four-week-old male and female C57BL/6J mice were purchased from DBL Co., Ltd. (Daejeon, Republic of Korea) and housed under controlled conditions (21 ± 2 °C, 12-h light/dark cycle) with ad libitum access to food and water. After a one-week acclimation period, five-week-old male and female mice were randomly assigned to three groups: Chow, HFD, and KBN2201. All animals were housed at five mice per cage, with one cage per group for each sex.

The normal chow diet was Teklad Irradiated Global 18% Protein Rodent Diet (2918; Teklad Diets, Inotiv, West Lafayette, IN, USA; energy density 3.1 kcal/g). The HFD provided 60% of total calories from fat (D12492; Saeron Bio, Cheonan-si, Republic of Korea; energy density 5.24 kcal/g). Both male and female groups were maintained on their respective diets for 12 weeks to induce diet-induced obesity. This model was used to evaluate the effects of KBN2201 on HFD-induced body weight gain, food intake, and appetite-related gut–brain markers.

All procedures were performed identically across sexes, and sex-stratified analyses were conducted. Male data are presented in the main figures, whereas female data were analyzed separately. The animal study was approved by the Institutional Animal Care and Use Committee of the Advanced Medical Bio Research Center (IACUC-2024-009) and conducted in accordance with institutional and national guidelines.

### 4.2. Drug Treatment

KBN2201, a small-molecule derivative with the molecular formula C_11_H_7_F_3_O_5_ and a molecular weight of 276.17 g/mol, was synthesized and supplied by KAISER Bio Ltd. (Gyeongsan, Republic of Korea). For oral administration, KBN2201 was first dissolved in dimethyl sulfoxide (DMSO) and then diluted in phosphate-buffered saline (PBS) to prepare a dosing solution containing 1% DMSO and 2 mg/mL KBN2201. Mice assigned to the KBN2201 group received the compound once daily by oral gavage at 20 mg/kg/day using a 20-gauge feeding needle. The dose of 20 mg/kg/day was selected based on previous in vivo studies of KBN2201, in which this oral dosing regimen produced biological efficacy without overt adverse effects [24,25]. This fixed-dose regimen was used as a proof-of-concept approach to evaluate whether continuous KBN2201 administration could attenuate HFD-induced obesity-associated phenotypes; therefore, the present study was not designed to determine the minimum effective dose or dose–response relationship. Vehicle-treated mice in the Chow and HFD groups were administered the same volume of 1% DMSO in PBS. The formulation and oral gavage procedures were adapted from our previous HFD study [48], with modification of the test compound and treatment duration. Treatment was initiated on the first day of diet exposure and was continued throughout the 12-week HFD-feeding period (Figure 1A). This dosing schedule was designed to evaluate the effects of continuous KBN2201 administration on HFD-induced body weight gain and feeding-related metabolic responses.

### 4.3. Physiological Measurements and Tissue Collection

Body weight and food intake were monitored throughout the 12-week experimental period. Food intake was assessed at the cage level by measuring food disappearance. Because mice were group-housed, food intake values represent cage-level repeated measurements rather than independent individual-level measurements. Daily food intake and caloric intake were therefore used to describe feeding patterns over time. A pre-weighed amount of diet was provided, and the remaining diet was collected and weighed at the indicated time points. Visible food fragments dispersed into the bedding were recovered as completely as possible and included in the measurement. Cage-level food intake was converted to average intake per mouse by dividing the total intake by the number of animals in each cage. To account for differences in energy density between the chow and HFD, food intake was also converted to energy intake using the manufacturer-provided energy density of each diet.

At the end of the 12-week treatment period, behavioral analyses were performed prior to tissue collection, as described below. One week after completion of behavioral testing, mice were euthanized according to the approved animal experimental protocol, and major organs and metabolic tissues were rapidly excised and weighed. eWAT, BAT, colon tissue, brain regions, TA muscle, and rectus femoris muscle were collected for further analysis. Portions of eWAT, BAT, colon tissue, TA muscle, and rectus femoris muscle were fixed in 4% paraformaldehyde for histological analysis. The remaining portions of eWAT, BAT, and colon tissue were snap-frozen and stored at −80 °C for molecular analysis. Brain regions associated with feeding regulation, including the hypothalamus and NTS-containing brainstem tissue, were rapidly dissected, frozen, and stored at −80 °C until RNA extraction. Tissue collection and processing were performed consistently across all experimental groups.

### 4.4. RNA Extraction and RT-qPCR

Total RNA was extracted from frozen colonic tissue, adipose tissue, and dissected brain regions, including the hypothalamus and NTS-containing brainstem tissue, using TRIzol reagent (Thermo Fisher Scientific, Waltham, MA, USA) according to the manufacturer’s protocol. Complementary DNA was synthesized from 1–5 μg of total RNA using Oligo-dT15 primer (TaKaRa Bio Inc., Kusatsu Shiga, Japan), PrimeScript™ Reverse Transcriptase (Cat. No. 2680A), and Recombinant RNase Inhibitor (TaKaRa Bio Inc., Kusatsu, Shiga, Japan). Quantitative real-time PCR was performed using TB Green Premix Ex Taq II (TaKaRa Bio Inc., Kusatsu, Shiga, Japan) on a QuantStudio 3 real-time PCR system (Thermo Fisher Scientific, Waltham, MA, USA). PCR amplification was performed under the following conditions: 50 °C for 2 min and 95 °C for 10 min, followed by 40 cycles of 95 °C for 15 s and 60 °C for 60 s. Melt curve analysis was performed to confirm amplification specificity. Genes related to feeding regulation, gut-derived anorexigenic signaling, lipid metabolism, thermogenesis, and inflammation were analyzed using primers listed in Table S1. *Gapdh* was used as the endogenous reference gene, and relative mRNA expression was calculated using the 2^−ΔΔCt^ method. The stability of *Gapdh* as the internal control gene was assessed by comparing its raw Ct values among experimental groups within each tissue analyzed. Data are summarized in Table S2 as the mean ± SD and coefficient of variation (CV), and group differences were evaluated using the Kruskal–Wallis test.

### 4.5. Histological Analysis

eWAT was fixed in 4% paraformaldehyde, embedded in paraffin, and sectioned at 4 μm thickness. Paraffin sections were processed through standard deparaffinization and rehydration procedures and stained with H&E. Adipocyte size was quantified from H&E-stained eWAT sections using Fiji/ImageJ software version 1.54. Non-overlapping fields were selected from comparable regions of each section, and adipocyte boundaries were manually delineated under the same workflow across all groups. The mean adipocyte area for each mouse was used as the statistical unit.

### 4.6. Behavioral Tests

Behavioral tests were performed after the 12-week dietary and treatment period in the following sequence: OFT, TST, and FST. To minimize the potential influence of fatigue, stress, or carryover effects, no more than one behavioral test was conducted per day, and mice were allowed at least 24 h of recovery between tests. The FST was performed last because it involves acute water exposure and is considered more physically stressful than the other behavioral assessments. Mice were acclimated to the behavioral testing room for at least 30 min before each test. All behavioral tests were recorded and analyzed using EthoVision XT software, version 11.5 (Noldus Information Technology, Wageningen, The Netherlands). The testing apparatus was cleaned between animals, and behavioral analyses were performed by an investigator blinded to group allocation.

Locomotor activity and anxiety-like behavior were assessed using the OFT. Each mouse was placed in the center of an open field arena and allowed to freely explore for 10 min. Total distance traveled, mean velocity, and time spent in the center and peripheral zones were analyzed. To reduce the influence of novelty-induced exploratory activity, data from the final 5 min of the session were used for quantitative analysis.

Depression-like behavior was assessed using the TST and FST. In the TST, mice were suspended by the tail using adhesive tape attached approximately 2 cm from the tail tip. Each mouse was tested for 6 min, and immobility time was quantified. In the forced swim test, mice were placed individually in a transparent cylinder filled with water maintained at 26 ± 1 °C. Each mouse was tested for 6 min, and immobility time during the final 4 min was analyzed. Immobility was defined as the absence of active escape-directed behavior, except for minimal movements required to maintain posture or keep the head above water. Only immobility episodes lasting longer than 1 s were included in the analysis.

### 4.7. Statistical Analysis

Data are presented as the mean ± SEM unless otherwise indicated. Statistical analyses were performed using GraphPad Prism 10 software (GraphPad Software, San Diego, CA, USA). Normality and homogeneity of variances were assessed using the Shapiro–Wilk test and Brown–Forsythe test, respectively. For single-time-point outcomes, including organ weight, adipocyte size, quantitative PCR, histological quantification, and behavioral parameters, one-way ANOVA followed by Tukey’s multiple comparisons test was used when the assumptions of normality and equal variance were met. When these assumptions were not satisfied, the Kruskal–Wallis test followed by Dunn’s multiple comparisons test was applied. Body weight changes over time were analyzed using two-way repeated-measures ANOVA with group and time as factors, followed by Tukey’s multiple comparisons test. Food intake and energy intake were assessed at the cage level. Because each sex had one cage per group, daily food intake values were considered repeated cage-level measurements and were presented descriptively without inferential statistical testing. Correlation analyses were performed to evaluate associations between colonic anorexigenic gene expression and central appetite-related gene expression. Pearson’s correlation analysis was used for normally distributed data, whereas Spearman’s rank correlation analysis was applied when normality was not satisfied. Statistical significance was set at *p* < 0.05.

## Data Availability

The datasets generated and analyzed in this study are available from the corresponding author upon reasonable request.

## Supplementary Materials

The following supporting information can be downloaded at: https://www.mdpi.com/article/10.3390/ijms27146155/s1.

## Abbreviations

The following abbreviations are used in this manuscript:

HFD	High-fat diet
PYY	Peptide YY
Y2R	Neuropeptide Y receptor type 2
NTS	Nucleus tractus solitarius
NPY	Neuropeptide Y
AgRP	Agouti-related peptide
POMC	Pro-opiomelanocortin
CART	Cocaine- and amphetamine-regulated transcript
GLP-1	Glucagon-like peptide-1
GIP	Glucose-dependent insulinotropic peptide
eWAT	Epididymal white adipose tissue
BAT	Brown adipose tissue
TA	Tibialis anterior
OFT	Open field test
FST	Forced swim test
TST	Tail suspension test
