# Supplementary material for: KBN2201 Attenuates High-Fat Diet-Induced Adipose Tissue Expansion and Body Weight Gain in Male Mice
Source: Int J Mol Sci. 2026 Jul 9;27(14):6155. doi: 10.3390/ijms27146155 (PMC13410170; doi:10.3390/ijms27146155)
Supplement: Supplementary file 1 [file ijms-27-06155-s001.zip › ijms-4390974-supplementary.pdf]

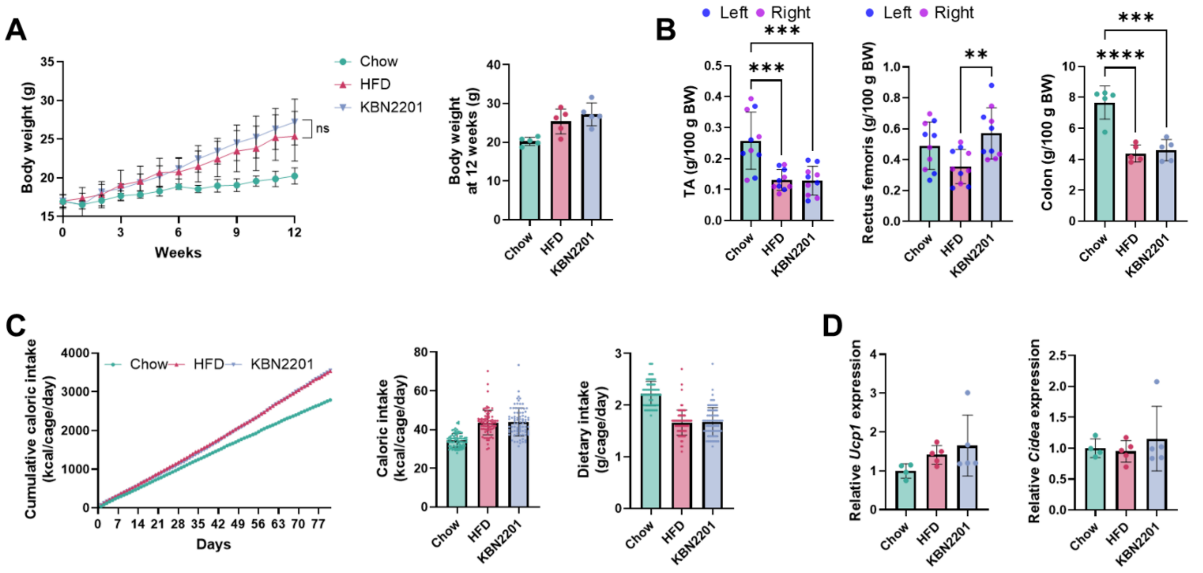


**Figure S1.** Supplementary analysis of body weight, tissue weight, food intake, and brown adipose tissue (BAT) thermogenic gene expression. (A) Body weight changes in female mice during the 12-week experimental period and final body weight at week 12. Body weight curves were analyzed using two-way repeated-measures ANOVA followed by Tukey's multiple comparisons test. No significant difference (ns) was detected between the HFD and KBN2201 groups throughout the 12-week period. (B) Tissue weight-to-body weight ratios of tibialis anterior (TA), rectus femoris, and colon in male mice, expressed as g/100 g body weight (BW). (C) Female cumulative caloric intake, daily cage-level caloric intake, and daily cage-level food intake. Food intake was measured at the cage level and is therefore presented as descriptive cage-level intake. (D) Relative mRNA expression of *Ucp1* and *Cidea* in male BAT. Data are presented as the mean ± SEM. Individual points represent biological replicates, except for cage-level food-intake data. Statistical significance is indicated for biological-replicate datasets only. $^{**}p < 0.01$, $^{***}p < 0.001$, and $^{****}p < 0.0001$.

**Table S1.** Primer sequences for RT-qPCR.

| Gene | Amplicon size (bp) | Forward primer (5'→3') | Reverse primer (5'→3') |
|---|---|---|---|
| *Npy* | 161 | ATG CTA GGT AAC AAG CGA ATG G | TGT CGC AGA GCG GAG TAG TAT |
| *Agrp* | 141 | ATG CTG ACT GCA ATG TTG CTG | CAG ACT TAG ACC TGG GAA CTC T |
| *Cartpt* | 103 | CCC GAG CCC TGG ACA TCT A | GCT TCG ATC TGC AAC ATA GCG |
| *Gcg* | 149 | TTA CTT TGT GGC TGG ATT GCT T | AGT GGC GTT TGT CTT CAT TCA |
| *Pyy* | 177 | ACG GTC GCA ATG CTG CTA AT | GAC ATC TCT TTT TCC ATA CCG CT |
| *Glp1r* | 117 | ACG GTG TCC CTC TCA GAG AC | ATC AAA GGT CCG GTT GCA GAA |
| *Npy2r* | 131 | GCC AGG GCA CAC TAC TCC TA | CTA CCC CTA GCA AGA TGA TGG A |
| *Ucp1* | 133 | AGGCTTCCAGTACCATTAGGT | CTGAGTGAGGCAAAGCTGATTT |
| *Srebf1* | 104 | GATGTGCGAACTGGACACAG | CATAGGGGGCGTCAAACAG |
| *Scd1* | 98 | TTCTTGCGATACACTCTGGTGC | CGGGATTGAATGTTCTTGTCGT |
| *Cidea* | 84 | TGACATTCATGGGATTGCAGAC | GGCCAGTTGTGATGACTAAGAC |
| *Ifng* | 182 | ATGAACGCTACACACTGCATC | CCATCCTTTTGCCAGTTCCTC |
| *Tgfb1* | 64 | AGCTGGTGAAACGGAAGCG | GCGAGCCTTAGTTTGGACAGG |
| *Il10* | 66 | ACAGCCGGGAAGACAATAACT | GCAGCTCTAGGAGCATGTGG |
| *Nos2* | 177 | ACATCGACCCGTCCACAGTAT | CAGAGGGGTAGGCTTGTCTC |
| *Tnf* | 89 | CAGGCGGTGCCTATGTCTC | CGATCACCCCGAAGTTCAGTAG |
| *Il1b* | 116 | GAAATGCCACCTTTTGACAGTG | CTGGATGCTCTCATCAGGACA |
| *Il6* | 76 | TAGTCCTTCCTACCCCAATTTCC | TTGGTCCTTAGCCACTCCTTC |
| *Gapdh* | 123 | AGG TCG GTG TGA ACG GAT TTG | TGT AGA CCA TGT AGT TGA GGT CA |

**Table S2.** Stability assessment of *Gapdh* raw Ct values across experimental groups in each tissue.

| **Tissue** | **Group** | **n** | **Gapdh, Ct (Mean ± SD)** | **CV (%)** | **Kruskal–Wallis (p-value)** |
|---|---|---|---|---|---|
| Colon | Chow | 4 | 14.9576 ± 0.1737 | 1.16 | 0.2206 |
| | HFD | 5 | 14.8496 ± 0.2226 | 1.50 | |
| | KBN2201 | 5 | 15.1626 ± 0.3092 | 2.04 | |
| WAT | Chow | 5 | 17.2803 ± 0.8215 | 4.75 | 0.1496 |
| | HFD | 5 | 17.6174 ± 0.2613 | 1.48 | |
| | KBN2201 | 4 | 17.9734 ± 0.8182 | 4.55 | |
| BAT | Chow | 4 | 15.2637 ± 0.2968 | 1.94 | 0.4441 |
| | HFD | 5 | 15.4733 ± 0.2204 | 1.42 | |
| | KBN2201 | 5 | 15.3723 ± 0.4363 | 2.84 | |
| NTS-containing brainstem | Chow | 5 | 15.5580 ± 0.1413 | 0.91 | 0.5273 |
| | HFD | 5 | 15.6110 ± 0.1143 | 0.73 | |
| | KBN2201 | 5 | 15.7738 ± 0.3339 | 2.12 | |
| Hypothalamus | Chow | 5 | 16.1713 ± 0.2061 | 1.27 | 0.1075 |
| | HFD | 5 | 16.0635 ± 0.1784 | 1.11 | |
| | KBN2201 | 5 | 15.8091 ± 0.2425 | 1.53 | |

Data are presented as the mean ± SD. CV was calculated as SD/mean × 100. p-values were calculated using the Kruskal–Wallis test for comparisons among the Chow, HFD, and KBN2201 groups within each tissue.
